# Antagonistic Role of CotG and CotH on Spore Germination and Coat Formation in *Bacillus subtilis*

**DOI:** 10.1371/journal.pone.0104900

**Published:** 2014-08-12

**Authors:** Anella Saggese, Veronica Scamardella, Teja Sirec, Giuseppina Cangiano, Rachele Isticato, Francesca Pane, Angela Amoresano, Ezio Ricca, Loredana Baccigalupi

**Affiliations:** 1 Department of Biology, Federico II University of Naples, Naples, Italy; 2 Department of Chemistry, Federico II University of Naples, Naples, Italy; University of Padova, Medical School, Italy

## Abstract

Spore formers are bacteria able to survive harsh environmental conditions by differentiating a specialized, highly resistant spore. In *Bacillus subtilis*, the model system for spore formers, the recently discovered crust and the proteinaceous coat are the external layers that surround the spore and contribute to its survival. The coat is formed by about seventy different proteins assembled and organized into three layers by the action of a subset of regulatory proteins, referred to as morphogenetic factors. CotH is a morphogenetic factor needed for the development of spores able to germinate efficiently and involved in the assembly of nine outer coat proteins, including CotG. Here we report that CotG has negative effects on spore germination and on the assembly of at least three outer coat proteins. Such negative action is exerted only in mutants lacking CotH, thus suggesting an antagonistic effect of the two proteins, with CotH counteracting the negative role of CotG.

## Introduction

Spore formers are Gram-positive bacteria belonging to different genera and including more than 1,000 species [1]. The common feature of these organisms is the ability to differentiate a spore, a dormant cell type that can survive for long periods in the absence of water and nutrients and resisting to a vast range of stresses (high temperature, dehydration, absence of nutrients, presence of toxic chemicals) [2]. When the environmental conditions ameliorates the spore germinates originating a cell able to grow and eventually sporulate [3]. Spore resistance to lytic enzymes and toxic chemicals is in part due to the presence of the spore coat, a multilayered structure composed by more than 70 proteins that surrounds the spore [4], [5]. Development of the mature spore is finely controlled through different mechanisms acting at various levels. The synthesis of coat proteins (Cot proteins) is regulated by a cascade of transcription factors controlling the timing of expression of their structural genes (*cot* genes) while coat assembly is controlled by a subset of Cot protein with a morfogenetic role [5]. Among the morphogenetic proteins, CotH plays a role in the assembly of at least 9 other coat components, including CotG, CotC/U and CotS, [6]–[9]. In addition, CotH contributes to the formation of spores able to germinate efficiently and to resist to lysozyme treatment [9]. CotH action is strictly connected with that of the major outer coat regulator CotE and mutant spores lacking both CotH and CotE germinate less efficiently and showed an increased sensitivity to lysozyme than single *cotE* null spores [9]. A recent report has shown that, when over-expressed, CotH bypasses the requirement for CotE, and suggests that CotE acts by localizing CotH on the spore coat and thus allowing its activity. In the presence of high CotH concentrations, due to the gene over-expression, CotH does not require CotE anymore and is able to drive the assembly of CotH-dependent proteins in a CotE-independent way [10].

The *cotH* structural gene is clustered with two other *cot* genes: *cotB*, transcribed in the same direction, and *cotG* divergently oriented with respect to *cotH*. A recent paper [11] has shown that the *cotH* promoter maps more than 800 bp upstream of its coding region, that this region is not translated and entirely contains the divergently transcribed *cotG* gene. A direct consequence of this peculiar chromosomal organization is that *cotG* insertion/deletion mutations so far analyzed [12], should also affect *cotH* expression leading to double *cotG cotH* mutants. If this is the case, then, the role of CotG has never been studied in an otherwise wild type strain and induces us to reconsider some previously reported results. Indeed, *cotG* spores have been previously reported as identical to isogenic wild type spores for both germination efficiency and lysozyme-resistance [12], while *cotH* spores have been shown to be about 35% less efficient than isogenic wild type spores upon induction of germination [8]. However, if an insertion-deletion within *cotG* impairs also the expression of *cotH* [11], those data imply that when both CotG and CotH are both lacking spores germinate normally but when only CotH is lacking spore germination is defective. In order to clarify the role CotG and its interaction with CotH, we first verified that CotH is not produced in a strain with an insertion/deletion mutation in *cotG* and then constructed for the first time a single *cotG* null mutant. The phenotypic analysis of the mutant spores is reported.

## Results and Discussion

### Construction of a *cotG* mutant

**Figure 1:**
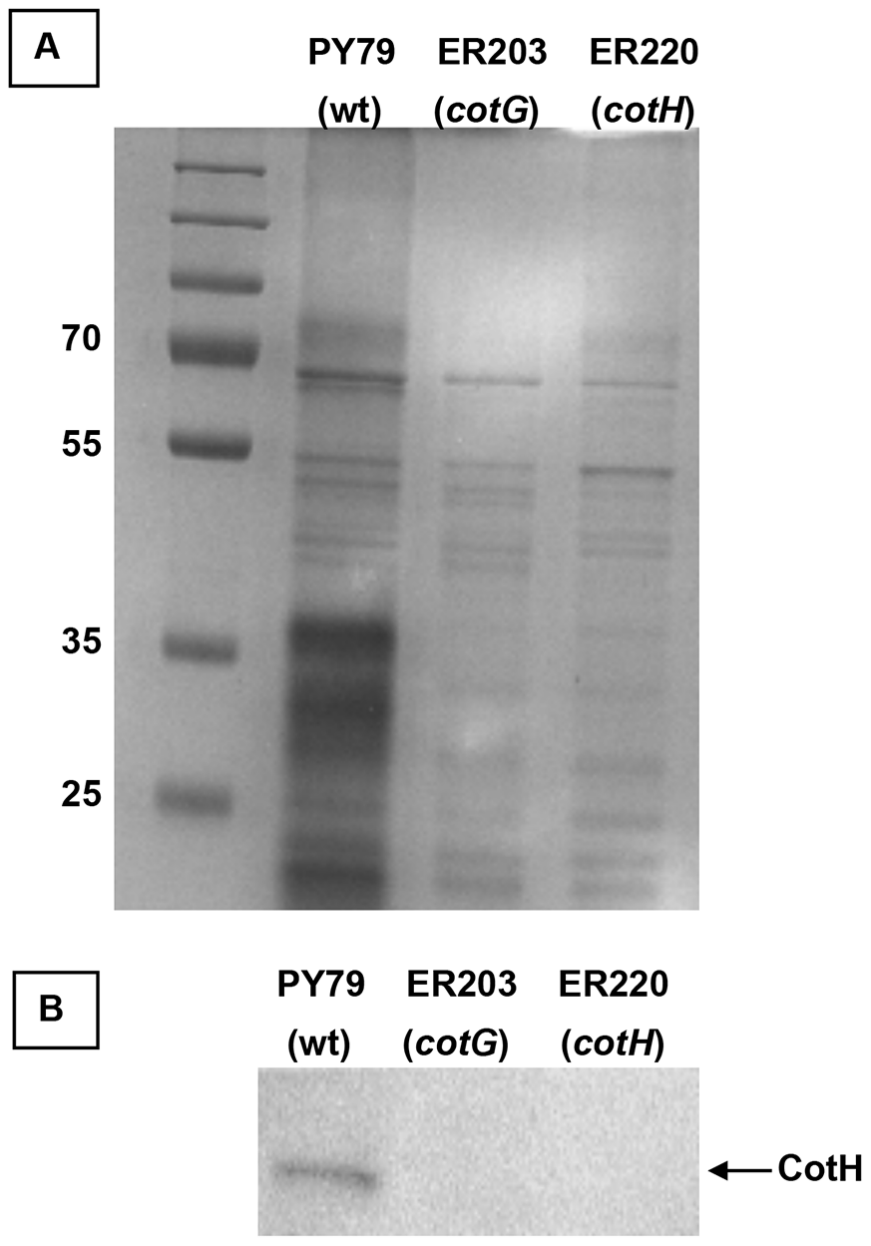
Production of CotH in a *cotG* null mutant. (A) SDS-PAGE fractionation of coat proteins from a wild type strain (PY79) and isogenic strains carrying null mutations in *cotG* (ER203) or in *cotH* (ER220). A molecular weight marker is also present and the size of relevant bands indicated. (B) Western blot with anti-CotH antibody of the same three strains analyzed in panel A. The arrow points to the CotH specific band.

To verify whether a strain with an insertion/deletion mutation in *cotG* produced CotH, coat proteins extracted from a wild type strain (PY79) and of two isogenic mutants in *cotG* (ER203) or in *cotH* (ER220) were compared. As previously reported [8], both mutants have on SDS-PAGE a strongly altered pattern of coat proteins with several minor differences characteristic of the two strains [8] (Fig. 1A). A western blot analysis with anti-CotH antibody of the coat proteins of the three strains confirmed that CotH is not produced in a strain with an insertion/deletion mutation in *cotG* (Fig. 1B).

**Figure 2:**
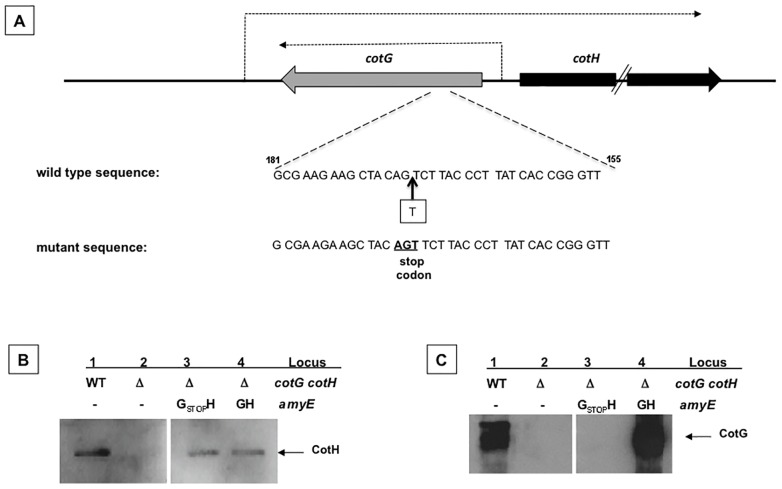
Construction of a single *cotG* mutant. (A) Thick gray and black arrows indicate the coding parts of *cotG* and *cotH*, respectively. Dashed arrow indicates the mRNA produced from the *cotG* and *cotH* promoters, as already reported. Site of insertion of the additional base in the *cotG* coding sequence (wild type sequence) that causes the formation of a premature stop codon (mutant sequence). Western blot analysis with anti-CotH (B) and anti-CotG (C) antibodies of proteins extracted by SDS treatment from wild type and isogenic mutant spores. The mutants genotype relative to the *cotG cotH* and *amyE* loci is indicated. Arrows point the CotH and CotG specific bands.

In order to obtain a *cotG* null mutation that does not affect *cotH* transcription, we introduced a single nucleotide in the *cotG* coding region by gene-soeing [13], thus causing the formation of a stop codon 21 bp downstream of the *cotG* translation start site (Fig. 2A). The entire *cotG_stop_cotH* region was PCR amplified, cloned into an integrative vector and inserted at the *amyE* locus on the *B. subtilis* chromosome of strain AZ603 carrying a deletion of the entire *cotG cotH* locus, yielding strain AZ604. An identical strategy was followed to PCR amplify, clone, integrate at the *amyE* locus and transfer into strain AZ603 a wild type copy of the *cotG cotH* region (AZ608). To verify the production of CotG and CotH in AZ604 (*ΔcotG ΔcotH amyE::cotG_stop_cotH*) and AZ608 (*ΔcotG ΔcotH amyE::cotGcotH*) western blots with anti-CotG or anti-CotH antibodies were performed. As shown in Fig. 2BC, the ectopic expression of a wild type copy of the *cotG cotH* region (lane 4 in both panels) in strain AZ603 complemented the deletion of the *cotG cotH* locus (lanes 2 in both panels). As expected, the ectopic expression of *cotG_stop_cotH* in strain AZ603 did not affect CotH production (panel B, lane 3) and did not produce CotG (panel C, lane 3).

### Role of CotG on spore germination and resistance to lysozyme

**Figure 3:**
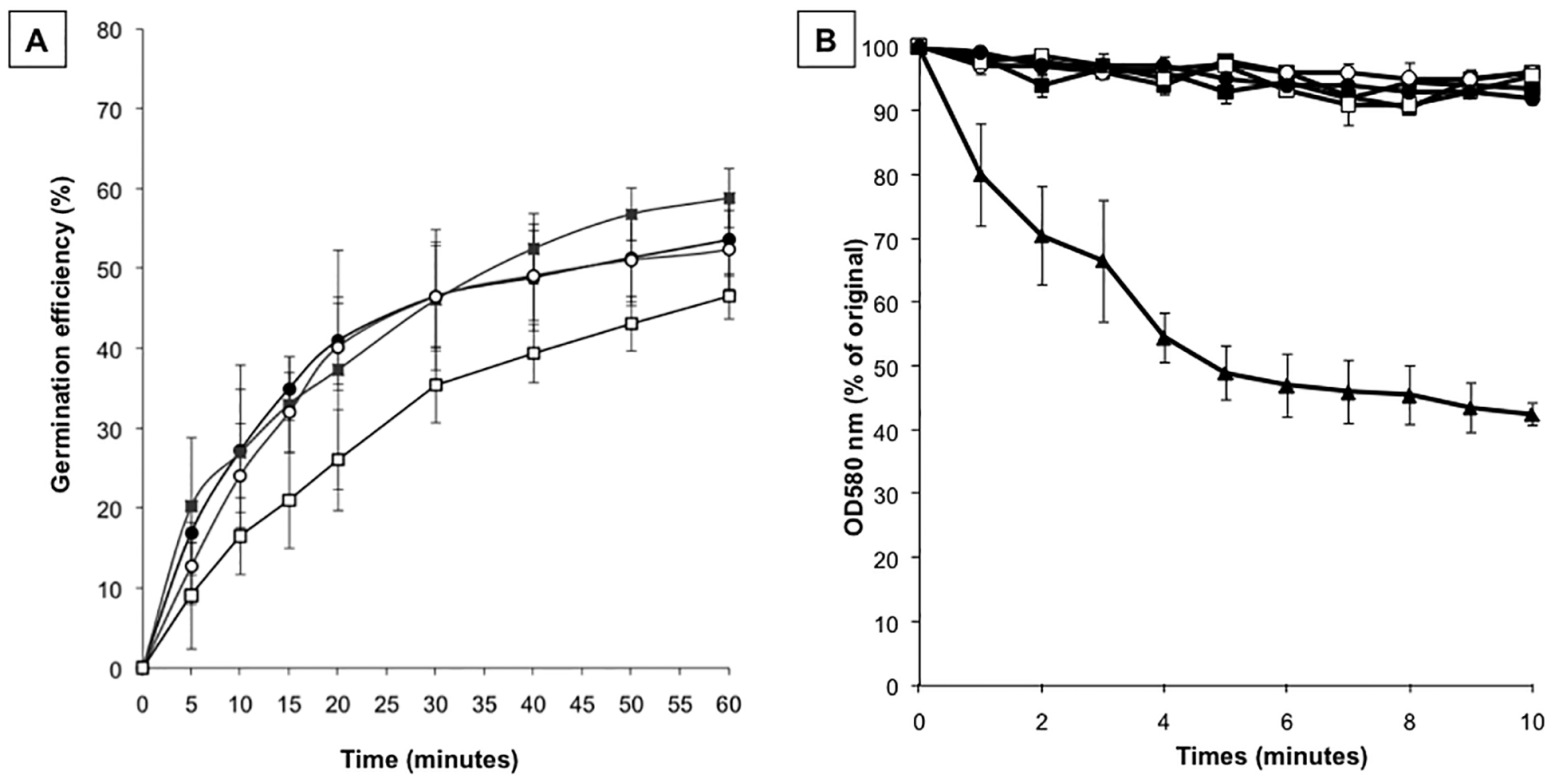
Germination efficiency and lysozyme-resistance assays. Spores derived from wild type (PY79, black circles), *cotG* null (AZ604, white circles), *cotH* null (ER220, white squares) and *cotGcotH* null (AZ603, black squares) were tested for germination efficiency (A) and for lysozime resistance (B). Germination was induced by Asn-GFK and measured as percentage of loss of optical density at 580 nm. Similar results were obtained by using L-Ala to induce germination. A *cotE* null strain (black triangles) known to be sensitive to lysozyme has been used as positive control during the lysozime treatment. Error bars are based on the standard deviation of 4 independent experiments.

We used the single *cotG* null mutant strain (AZ604) to analyze the efficiency of germination and the resistance to lysozyme. Together with AZ604 we considered for our analysis spores of three other isogenic strains: a wild type (PY79) containing both CotG and CotH [8], [12], *cotH* null (ER220) containing only CotG [7] and *cotH cotG* null (AZ603) lacking both proteins. As shown in Fig. 3A, AZ604 spores (*cotG*) showed an efficiency of germination identical to that of wild type spores (white and gray circles in the figure). As previously reported [8], spores of the *cotH* null strain were slightly less efficient in germination than wild type spores (white squares in Fig. 3A). With spores of strain AZ603 (*cotG cotH*) the germination efficiency was restored to wild type levels (black squares in Fig. 3A). These results indicate that the germination defect observed with spore lacking only CotH was rescued in spores lacking both CotH and CotG. As a consequence they suggest that the germination impairment is not directly due to the absence of CotH as previously believed [8] but instead to the presence of CotG in a *cotH* null background. This finding also suggest a protective role for CotH in counteracting the CotG negative effect. The same four strains were also used to analyze the spore resistance to lysozyme and were all identical to wild type spores (Fig. 3B).

### Role of CotG on coat protein assembly

**Figure 4:**
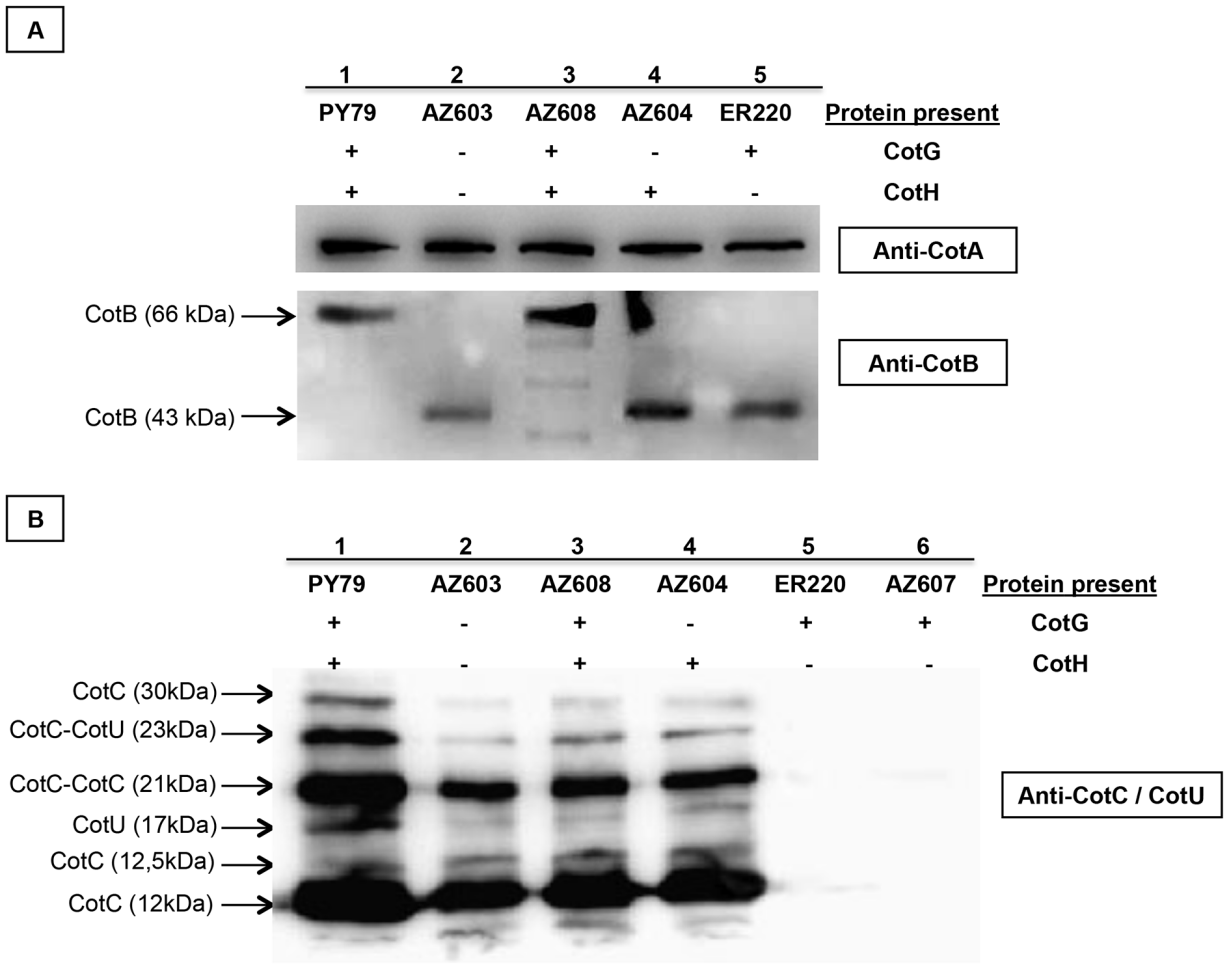
Western blot analysis. Western blot analysis of proteins extracted from mature spores of wild type (PY79, lane 1), *ΔcotGΔcotH* (AZ603, lane 2), *ΔcotGΔcotH amyE::cotGcotH* (AZ608, lane 3), *ΔcotGΔcotH amyE::cotG_stop_cotH* (AZ604, lane 4), *cotH::spc* (ER220, lane 5) and *ΔcotGΔcotH amyE::cotG* (AZ607, lane 6 of panel B) strains. For CotA and CotB detection (panel A) the proteins have been extracted by SDS treatment while for CotC and CotU detection (panel B) the NaOH treatment has been used. Proteins (25 µg) were reacted with CotA, CotB and CotC specific rabbit antibodies and then with peroxidase-conjugated secondary antibodies and visualized by the Pierce method. The estimated size of CotB, CotC and CotU is indicated.

We then analyzed the assembly of various coat proteins in the presence and in the absence of CotG and/or CotH. For our analysis we compared by western blot a wild type strain (PY79) and isogenic strains with an insertion/deletion in *cotH* (ER220, *cotH::spc*), or deleted of the entire *cotH cotG* locus (AZ603) and expressing either a wild type (AZ608) or a *cotG* mutant (AZ604) copy of the *cotH cotG* locus. As shown in Fig. 4, our analysis confirmed that levels of CotA (a CotH-independent protein) is not affected by CotH and/or CotG and that CotB maturation is dependent on the presence of both CotG and CotH [14]. Indeed, in spores of strains lacking CotG or CotH or both, CotB is assembled within the coat in its immature 43 kDa form. Only when both CotG and CotH are present the mature protein of 66 kDa is formed (Fig. 4A).

CotC and CotU are two CotH-dependent proteins that are homologous and recognized by both anti-CotC and anti-CotU antibodies [15]. CotC is present within the spore coat as a monomer (12 kDa), homodimer (21 kDa) and as two additional forms of 12.5 and 30 kDa [16]. CotU is found as a 17 kDa monomer [15] and as a heterodimer with CotC of 23 kDa [17]. As expected, all the CotC/CotU forms are found when both CotG and CotH are present (Fig. 4B, lanes 1 and 3) and none of them is observed when CotH is not expressed (Fig. 4B, lane 5). However, when both CotH and CotG are lacking (Fig. 4B, lane 2) as well as when only CotG is lacking (Fig. 4B, lane 4) all CotC/CotU proteins are normally assembled on the spore. These data indicate that, as for the germination phenotype, CotG has a negative role on CotC/CotU assembly and that its role is counteracted by CotH. To confirm the negative effect of CotG in a *cotH* background, we inserted an ectopic copy of *cotG* allele at *amyE* locus in the double *cotGcotH* mutant and also in this case all the CotC/CotU forms are no more assembled in the coat (Fig. 4B, lane 6).

**Figure 5:**
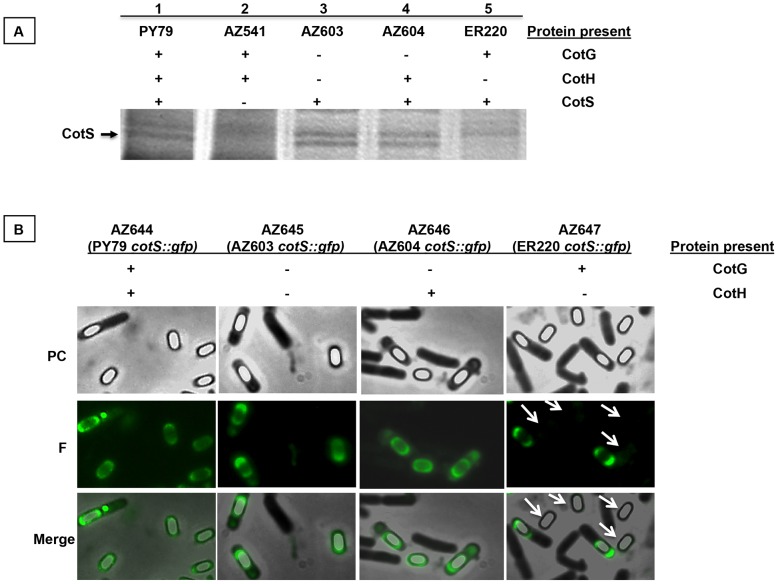
SDS-PAGE and Fluorescence analysis. (A) Proteins released after treatment with SDS of spores of the indicated strains were fractionated on a 12,5% polyacrilamide gel. The arrow indicates the 41 kDa band correspoding to CotS (18). The gel was stained with Coomassie brilliant blue. (B) Strains carrying the *cotS::gfp* fusion were analyzed by phase-contrast (PC) and fluorescence (F) microscopy. The bottom panel reports a merge of the two images. Exposure time was 588 ms in all cases.

CotS is 41 kDa, *cotH*-dependent spore coat protein [18], clearly identified by SDS-PAGE and western blot [19]. As shown in Fig. 5A, a protein absent in the *cotS* null mutant (AZ541, lane 2), is not present in the *cotH* mutant (ER220, lane 5) but is present in both the single *cotG* mutant (AZ604, lane 4) and in the double *cotH cotG* mutant (AZ603, lane 3). To confirm this SDS-PAGE analysis we constructed a *cotS::gfp* fusion and integrated it on the chromosome of a wild type strain (PY79). By chromosomal DNA-mediated transformation we then moved the fusion into strains AZ603 (*ΔcotG ΔcotH*), AZ604 (*ΔcotG ΔcotH amyE::cotG_stop_cotH*) and ER220 (*cotH::spc*) and analyzed all resulting strains by fluorescence microscopy. A fluorescence signal was observed around mature and forming spores in a wild type strain and in isogenic strains lacking both CotH and CotG (AZ603) or lacking only CotG (AZ604) (Fig. 5B). However, when CotG is present and CotH is lacking (ER220) [7] a fluorescence signal was observed around forming spores but never around mature, free spores (Fig. 5B). This result is in agreement with the SDS-PAGE of Fig. 5A, performed with proteins extracted from mature spores, and indicates that, also for CotS assembly, CotG has a negative role antagonized by CotH.

### On the nature of CotG-CotH interaction

**Figure 6:**
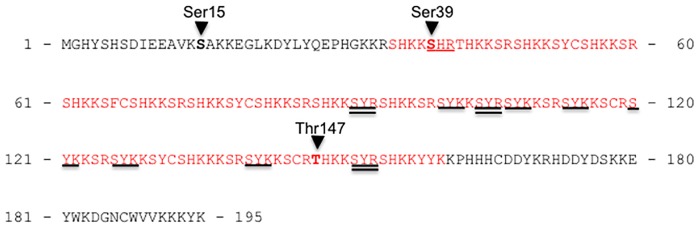
CotG and phosphorylation sites. Results of a mass spectrometry analysis of peptides derived from trypsine digestion of CotG are reported. Unambiguosly identified sites of phosphorylation are indicated. Tripeptides containing a phosphate moiety are underlined; the random coiled tandem repeats region is in red.

The nature of the antagonistic action of CotH on CotG negative role, suggested by results of Fig. 3, 4 and 5, is not clear. However some hints come from a recent bioinformatic analysis that has identified CotH as a putative kinase [20]. In addition, another previous report has shown that a *B. anthracis* protein with some similarities with CotG of *B. subtilis* is highly phosphorylated [21]. These literature data induced us to hypothesize that CotH is a kinase and CotG one of its substrates. To partially support this hypothesis we performed a mass spectrometry analysis of CotG. Coat proteins extracted from wild type spores were fractionated on SDS polyacrylamide gel and a region of the gel containing CotG used to reduce, alkylate and digest the proteins *in situ* with trypsin (see Material and Methods). The peptide mixture was divided in two aliquots and submitted to MALDIMS and nanoLCMSMS analyses and then directly analyzed by nanoHPLC-chip MS/MS. Due to the low resolution of the SDS-PAGE, more than one protein was identified in the same region of the gel but CotG exhibited the highest MASCOT score (not shown). Several phosphorylation sites were identified within CotG, some detected in the MALDIMS runs and some by a manual interpretation of the MS/MS spectra (Table S2 in File S1). Fig. 6 reports a summary of the phosphorylation sites identified in CotG. The occurrence of phosphorylation sites at level of Ser15, Ser39 and Thr147 was unambiguous and suggests that a kinases belonging to Serine-threonine kinase family is involved in CotG modification. Other phosphorylation sites occurred in amino acid sequences repeated several times within the CotG central region (for example, the tripeptides SYK underlined or SYR double-underlined in Fig. 6), thus impairing the exact localization of the modifications. Although we cannot definitely conclude that all of the underlined and double-underlined tripeptides are phosphorylated, the absence of the same tryptic fragments among the unmodified peptides strongly suggests that most, if not all of them are phosphorylated and that serine, always present in those tripeptides, is the most probable amino acid interested by the post-translational modification.

While Ser15 is in the N-terminal part of CotG, Ser39, Thr147 and all the other possible sites of phosphorylation are located in the repeated central region (Fig. 6 and Table S2 in File S1). This region is composed by random coiled repeats [11], each containing serine residues surrounded by positively charged amino acids (Fig. 6). In a bioinformatic analysis of known phosphorylated proteins [22] all these features have been indicated as typical of intrinsically disordered structures and have been identified as predictor of phosphorylation substrates.

In a *cotH* mutant CotG is not present around both the mature and the forming spore [8] but accumulates in the mother cell compartment of the sporulating cell [7]. However, its peculiar structure has so far impaired CotG isolation from the mother cell compartment of sporulating *B. subtilis* cells as well as from a heterologous host (*E. coli*), therefore not allowing further analysis. Although additional experiments, beyond the aims of this manuscript, will be needed to confirm that CotH is a kinase and CotG one of its substrates, we speculate that in a wild type strain CotG would be mainly present in a phosphorylated form and that, in this form, it plays its structural role as a coat component. In a *cotH* mutant, we predict that CotG would not be phosphorylated and have a negative effect on the assembly of some coat proteins and on spore germination.

## Conclusions

Because of the peculiar chromosomal organization of the *cotG cotH* locus [11], in a *cotG* null mutant also the expression of the *cotH* gene is impaired and, as a consequence, the presumed *cotG* mutant is a double mutant lacking both CotG and CotH. In this work we constructed for the first time a *cotG* null mutant in which CotH is produced. A phenotypic analysis of this mutant has shown that it does not differ significantly from the isogenic wild type strain but has also shown that phenotypes previously attributed to the lack of CotH are only observed when in the *cotH* strain is present CotG. When both CotH and CotG are absent the defects observed in the single *cotH* mutant are completely restored and the double mutant is indistinguishable from the isogenic wild type strain. This is the case of the germination defect of *cotH* spores that is rescued in a *cotG cotH* double mutant; is the case of CotC/U and CotS assembly within the coat. CotG has a peculiar primary structure: it has several repeats in its central part and has a high positive charge (pI 10.26). In a wild type strain CotG is highly phosphorylated and this post-translational modification is probably important to neutralize the positive charges and, consequently to guarantee protein stability and ability to interact with other coat components. The kinase responsible of this modification has not been identified yet. A recent bioinformatic data has indicated that CotH has some homology with eukaryotic Ser-Thr kinases [20] and our results functionally linking CotG to CotH, point to CotH as the kinase responsible of CotG phosphorylation. Future site-directed mutagenesis experiments will be needed to support this hypothesis.

## Methods

### Bacterial strains and transformation

*B. subtilis* strains are listed in Table 1. Plasmid amplification for nucleotide sequencing, subcloning experiments, and transformation of *E. coli* competent cells were performed with *Escherichia coli* strain DH5α [23]. Bacterial strains were transformed by previously described procedures: CaCl_2_-mediated transformation of *E. coli* competent cells [23] and two-step transformation of *B. subtilis* [24].

**Table 1:** *Bacillus subtilis* strains used in this study.

Strain	Relevant genotype	Reference
PY79	wild type	[32]
ER220	*cotH::spec*	[8]
AZ541	*cotS::cm*	[33]
AZ603	*ΔcotG ΔcotH*:*:neo*	This work
AZ604	*ΔcotG ΔcotH::neo amyE::cotG_stop_cotH*	This work
AZ608	*ΔcotG ΔcotH::neo amyE::cotGcotH*	This work
AZ607	*ΔcotG ΔcotH::neo amyE::cotG*	This work
AZ644	*cotS::gfp*	This work
AZ645	*ΔcotG ΔcotH::neo cotS::gfp*	This work
AZ646	*ΔcotG ΔcotH*::*neo amyE*::*cotG_stop_cotH cotS::gfp*	This work
AZ647	*cotS::gfp cotH::spec*	This work

### Genetic and molecular procedures

Isolation of plasmids, restriction digestion and ligation of DNA, were carried out by standard methods [23]. Chromosomal DNA from *B. subtilis* was isolated as described elsewhere [24].

### Deletion of the *cotG cotH* locus

The *cotG cotH* locus was entirely deleted and substituted by a neomycin-resistance (*neo*) gene cassette. Chromosomal DNA of strain PY79 was used as a template and oligonucleotide pairs Del3-H18 and H29-B-anti (Table S1 in File S1) were used to prime the PCR amplification of two DNA fragments of 361 bp and 704 bp, respectively located upstream and downstream of the *cotH* gene. The two DNA fragments were separately cloned in the pBEST501 vector [25] at 5′ or 3′ ends of the *neo* gene. The resulting plasmid, pVS6, was then linearized by restriction digestion with *Sca*I and used to transform competent cells of the PY79 strain of *B. subtilis*. Replacement of the *cotH cotG* locus on the chromosome with the *neo* gene occurred by double cross-over between homologous DNA sequences originating strain AZ603 (*ΔcotG ΔcotH*) and was verified by PCR.

### Construction of a single *cotG* mutant

The entire *cotH cotG* locus was PCR amplified using oligonucleotides Del5 and H28 (Table S1 in File S1) to prime the reaction and PY79 chromosomal DNA as a template. The resulting DNA fragment was cloned into plasmid pDG364 [24], yielding plasmid pVS8. To insert a single nucleotide within the cotG coding part (at position +22, considering as +1 the first nucleotide of the first *cotG* codon) we used a *gene soeing* approach [13]. Two partially overlapping DNA fragments were PCR amplified priming the reaction with oligonucleotide pairs Gstop/Del5 (743 bp) and Gstop-anti/H (317 bp) (Table S1 in File S1) and using chromosomal DNA of PY79 as a template. The obtained PCR products were used as templates to prime a third linear PCR of 7 cycles using only the external primers Del5 and H (Table S1 in File S1). The single-strand products thus obtained were mixed and used to perform a standard PCR program of 20 cycles that led to their cohesion. The recombinant fragment was cloned in pGemT easy vector (Promega) and controlled by sequencing to confirm the presence of the point mutation resulting in the substitution of the 8th *cotG* codon with a stop codon. The mutant *cotG* allele (here called *cotG_stop_*) was digested with *Bam*HI-*Bgl*II and cloned into pVS8 to replace the wild type *cotG* allele, yielding plasmid pVS7. Both plasmids pVS7 (carrying the *cotG_stop_cotH* locus) and pVS8 (carrying the wild type *cotG cotH* locus) were separately used to transform competent cells of AZ603 (*ΔcotG ΔcotH*). The occurrence of a single reciprocal (Campbell-like) recombination event between homologous DNA on the plasmids and on the chromosome (*amyE* locus) was verified by PCR.

### Ectopic expression of cotG

The entire *cotG* gene was PCR amplified priming the reaction with oligonucleotide pairs G22 and H19 (774 bp), cloned in pGEM-T Easy vector (Promega), controlled by sequencing and transferred into the integrative vector pDG364 [24] using *EcoR*I and *Bam*HI restriction sites.

The plasmid was used to transform the double mutant AZ603 (*ΔcotG ΔcotH*). The occurrence of a single reciprocal (Campbell-like) recombination event between homologous DNA sequences present on the plasmid and on the chromosome (*amyE* locus) was verified by PCR and yielded strain AZ607 (*ΔcotG ΔcotH, amyE::cotG*).

### Construction of *cotS::gfp* fusion

The *gfp mut3a* gene, encoding the green fluorescent protein (GFP) [26] was PCR amplified using plasmid pAD123 (Bacillus Genetic Stock Center, BGSC, www.bgsc.org) as a template and priming the reaction with oligonucleotides GFPfor and GFPrev (Table S1 in File S1). The *gfp mut3a* gene was cloned in pGEM-T Easy vector (Promega), controlled by sequencing and transferred into the integrative vector pER19 [27] using *Pst*I and *Bam*HI restriction sites. The region containing the entire *cotS* gene except the stop codon, was PCR amplified using chromosomal DNA of strain PY79 as a template and priming the reaction with oligonucleotides cotS-for and cotS-rev (table S1 in File S1), and cloned in frame with *gfp* using the *Sph*I restriction site located at 5′ end of *gfp*. The resulting plasmid pcotS-gfp was used to transform competent cells of strain PY79. The occurrence of a single reciprocal (Campbell-like) recombination event between homologous DNA sequences present on the plasmid and on the chromosome (*cotS* locus) yielded strain AZ644 (*cotS::gfp*) was verified by PCR. Chromosomal DNA of strain AZ644 was then used to transfer the *cotS-gfp* fusion into strains AZ603 *(ΔcotG ΔcotH*), AZ604 (*cotG_stop_*) and ER220 (*cotH::spec*), yielding respectively AZ645 (*ΔcotG ΔccotH cotS::gfp*), AZ646 (*cotG_stop_ cotS::gfp*), AZ647 (*cotH::spec cotS::gfp*). Fluorescence microscopy analysis was performed with an Olympus BX51 fluorescence microscope using a Fluorescein-Isothiocyanate (FITC) filter as previously reported [28]. Typical acquisition times were 588 ms and the Images were captured using a Olympus DP70 digital camera and processed.

### Spore purification, extraction of spore coat proteins and western blot analysis

Sporulation was induced by exhaustion by growing cells in DSM (Difco Sporulation Medium) as described elsewhere [24]. After a 30 hours of incubation at 37°C, spores were collected, washed four times, and purified as described by Nicholson and Setlow [29] using overnight incubation in H_2_O at 4°C to lyse residual sporangial cells. Spore coat proteins were extracted from a suspension of spores by SDS-dithiothreitol (DTT) [24], or NaOH [29] treatment as previously described. The concentration of extracted proteins was determined by using Bio-Rad DC protein assay kit (Bio-Rad), and 20 µg of total spore coat proteins were fractionated on 12,5% SDS polyacrylamide gels and electrotransferred to nitrocellulose filters (Bio-Rad) for Western blot analysis following standard procedures. CotH-, CotA-, CotC-, CotB- and CotG-specific antibodies were used at a working dilutions of 1∶150 for CotH detection and 1∶7000 for CotA, CotC, CotB and CotG detection. Then an horseradish peroxidase (HRP)-conjugated anti-rabbit secondary antibody was used (Santa Cruz). Western blot filters were visualized by the SuperSignal West Pico chemiluminescence (Pierce) method as specified by the manufacturer.

### Germination efficiency and lysozyme resistance

Purified spores were heat activated as previously described [24] and diluted in 10 mM Tris-HCl (pH 8.0) buffer containing 1 mM glucose, 1 mM fructose, and 10 mM KCl. After 15 min at 37°C, germination was induced by adding 10 mM L-alanine or 10 mM L-asparagine and the optical density at 580 nm was measured at 5-min intervals for 60 minutes [24].

Sensitivity to lysozyme was measured as described by Zheng *et al.* [30]. Spores were prepared as previously described [24], omitting the lysozyme step and eliminating vegetative cells by heat treatment (10 min at 80°C). Purified spores were then suspended in 10 mM Tris-HCl (pH 7.0) buffer containing lysozyme (50 mg/ml), and the decrease in optical density was monitored at 595 nm at 1-min intervals for 10 min. Spore viability was measured after 30 min as CFU on TY agar plates.

### In situ digestion and mass spectral analyses

Protein bands corresponding to CotG were excised from the gel and destained by repetitive washes with 0.1 M NH_4_-HCO_3_ pH 7.5 and acetonitrile. Samples were then submitted to in situ trypsin digestion and analyzed by MALDI mass spectrometry and LCMSMS as previously described [31]. The acquired MS/MS spectra were transformed in *mzData* (.XML) format and used for protein identification with a licensed version of MASCOT software (www.matrixscience.com) version 2.4.0. Raw data from nanoLC-MS/MS analysis were used to query the NCBInr database NCBInr 20121120 (21,582,400 sequences; 7,401,135,489 residues). Mascot search parameters were: trypsin as enzyme; 3, as allowed number of missed cleavage; carboamidomethyl as fixed modification; oxidation of methionine; phosphorylation of serine/threonine/tyrosine; pyro-Glu N-term Q as variable modifications; 10 ppm MS tolerance and 0.6 Da MS/MS tolerance; peptide charge from +2 to +3. Peptide score threshold provided from MASCOT software to evaluate quality of matches for MS/MS data was 25.Spectra with MASCOT score of <25 having low quality were rejected.

## Supporting Information

File S1 **Table S1: list of oligonucleotides used in this study. Table S2:** Mass spectral analyses of CotG trypsin digest.
(DOCX)
