# Supplementary material for: Antagonistic Role of CotG and CotH on Spore Germination and Coat Formation in Bacillus subtilis
Source: PLoS One. 2014 Aug 12;9(8):e104900. doi: 10.1371/journal.pone.0104900 (PMC4130616; doi:10.1371/journal.pone.0104900)
Supplement: File S1 — Table S1: list of oligonucleotides used in this study. Table S2: Mass spectral analyses of CotG trypsin digest. (DOCX) [file pone.0104900.s001.docx]

**Table S1.** **Oligonucleotides used in this study**

| Primer | Sequence 5' - 3'^(a)^ | Restriction  site | Position of annealing^(b)^ |
| --- | --- | --- | --- |
| Gstop | GCTTCTTCGATGTCAAGAATGGGAATAG |  | -175/-147 **(***cotH*) |
| Gstop-anti | CTATTCCCATTCTTGACATCGAAGAAGC |  | -147/-175 **(***cotH*) |
| Del5 | ggatccGCCTTTATCGTTAGGAT | BamHI | -884/-867 **(***cotH*) |
| H | cgcggatccgcgccggaattcAGCGATATCAATATCCAG | BamHIEcoRI | +126/+144 **(***cotH*) |
| H28 | gcatgcAATTCAATAGCCTAATTGTC | SphI | +1140/+1120 **(***cotH*) |
| H29 | ctgcagGCCGGATGTGATCTGCGAG | PstI | +1019/+1038 **(***cotH*) |
| B-anti | aagcttCGTCGGCATTATCTACAAGG | HindIII | +1814/+1794 **(***cotH*) |
| Del3 | ggatccCAAATTCTCCGTTCTCC | BamHI | -1145/-1128 **(***cotH*) |
| H18 | ggatccATTTGCCCTGTATTAGATATATG | BamHI | -805/-828 **(***cotH*) |
| G22 | TTCCGTACCTCCGCCGGCAGCC |  | +610/+632 (*cotG*) |
| H19 | ggatccCCATAATCCTCCTTACAAATT | BamH1 | -142/-121 (cotG) |
| CotS-for | gcatgcATGCCGAACGTATCAATG | SphI | -313/-295 **(***cotS*) |
| CotS-rev | gcatgcATTCGCCTCCCGATACG | SphI | +883/+900 (*cotS*) |
| GFP for | ctgcagATGAGTAAAGGAGAAGAAC | PstI | +1/+19 (*gfp*) |
| GFP rev | ggatccTTATTTGTATAGTTCATCCATGCC | BamHI | +694 /+717 **(***gfp*) |

^a^ Capital and lowercase letters indicate bases of DNA and of an unpaired tail carrying a restriction site (underlined).

^b^ Considering as +1 the first base of the first codon in *cotH*, *cotS, cotG* or *gfp* as indicated in brackets.

**Table S2.** **Mass spectral analyses of CotG trypsin digest**

| Observed m/z value | Post-translational Modification | Aminoacid Position | Sequence | Mass spectral technique |
| --- | --- | --- | --- | --- |
| 473.61 | - | 158-160 | YYK | MALDI |
| 477.43 | phosphorilation | 100-102 | SYK | MALDI |
| 477.43 | phosphorilation | 107-109 | SYK | MALDI |
| 477.43 | phosphorilation | 113-115 | SYK | MALDI |
| 477.43 | phosphorilation | 120-122 | SYK | MALDI |
| 477.43 | phosphorilation | 126-128 | SYK | MALDI |
| 477.43 | phosphorilation | 140-142 | SYK | MALDI |
| 479.1 | phosphorilation | 39-41 | SHR | MALDI |
| 505.26 | phosphorilation | 91-93 | SYR | MALDI |
| 505.26 | phosphorilation | 104-106 | SYR | MALDI |
| 505.26 | phosphorilation | 151-153 | SYR | MALDI |
| 518.3 | - | 136-139 | KKSR | MALDI |
| 720.24 | phosphorilation | 98-102 | SRSYK | MALDI |
| 720.24 | phosphorilation | 111-115 | SRSYK | MALDI |
| 720.24 | phosphorilation | 124-128 | SRSYK | MALDI |
| 720.24 | phosphorilation | 138-142 | SRSYK | MALDI |
| 893.42 | - | 64-70 | KSFCSHK | MALDI |
| 893.42 | - | 64-71 | SFCSHKK | MALDI |
| 893.42 | - | 38-44 | KSHRTHK | MALDI |
| 893.42 | - | 39-45 | SHRTHKK | MALDI |
| 909.16 | - | 51-57 | KSYCSHK | MALDI |
| 909.16 | - | 77-83 | KSYCSHK | MALDI |
| 909.16 | - | 129-135 | KSYCSHK | MALDI |
| 909.16 | - | 52-58 | SYCSHKK | MALDI |
| 909.16 | - | 78-84 | SYCSHKK | MALDI |
| 909.16 | - | 130-136 | SYCSHKK | MALDI |
| 1007.44 | - | 172-179 | HDDYDSKK | MALDI |
| 1676.42 | - | 19-32 | EGLKDYLYQEPHGK | MALDI |
| 976.45 | - | 184-191 | [DGNCWVVK](file:///C:\\Documents%20and%20Settings\\Chiara\\Impostazioni%20locali\\Temporary%20Internet%20Files\\peptide_view.pl?file=..\\data\\20130114\\F002064.dat&query=524&hit=1&index=gi\|16080660&db_idx=1&px=1&section=5&ave_thresh=25&_ignoreionsscorebelow=0&report=0&_sigthreshold=0.05&_msresflags=1089&_msresflags2=2&percolate=-1&percolate_rt=0&_minpeplen=7) | LCMSMS |
| 1104.54 | - | 184-192 | DGNCWVVKK | LCMSMS |
| 1582.72 | - | 180-191 | EYWKDGNCWVVK | LCMSMS |
| 1675.81 | - | 19-32 | EGLKDYLYQEPHGK | LCMSMS |
| 1675.82 | - | 19-32 | EGLKDYLYQEPHGK | LCMSMS |
| 710.82 | - | 180-192 | EYWKDGNCWVVKK | LCMSMS |
| 1710.82 | - | 179-191 | KEYWKDGNCWVVK | LCMSMS |
| 1803.92 | - | 18-32 | [KEGLKDYLYQEPHGK](file:///C:\\Documents%20and%20Settings\\Chiara\\Impostazioni%20locali\\Temporary%20Internet%20Files\\peptide_view.pl?file=..\\data\\20130114\\F002064.dat&query=1419&hit=1&index=gi\|16080660&db_idx=1&px=1&section=5&ave_thresh=25&_ignoreionsscorebelow=0&report=0&_sigthreshold=0.05&_msresflags=1089&_msresflags2=2&percolate=-1&percolate_rt=0&_minpeplen=7) | LCMSMS |
| 1964.88 | phosphorilation | 2-18 | GHYSHSDIEEAVKSAKK | LCMSMS |
| 2170.05 | phosphorilation | 15-32 | [SAKKEGLKDYLYQEPHGK](file:///C:\\Documents%20and%20Settings\\Chiara\\Impostazioni%20locali\\Temporary%20Internet%20Files\\peptide_view.pl?file=..\\data\\20130114\\F002064.dat&query=907&hit=1&index=gi\|16080660&db_idx=1&px=1&section=5&ave_thresh=25&_ignoreionsscorebelow=0&report=0&_sigthreshold=0.05&_msresflags=1089&_msresflags2=2&percolate=-1&percolate_rt=0&_minpeplen=7) | LCMSMS |
| 2571.15 | - | 172-191 | HDDYDSKKEYWKDGNCWVVK | LCMSMS |
